# Energy-Resolving Time-of-Flight Mass Spectrometry for Bulk Plasma Analysis

**DOI:** 10.1021/jasms.4c00140

**Published:** 2024-07-11

**Authors:** Malte Watzek, Patrick Sturm, Carsten Stoermer, Abdelhak Bensaoula, Thomas Nelis, Caroline Hain

**Affiliations:** † TOFWERK AG, Schorenstrasse 39, 3645 Thun, Switzerland; ‡ Institute for Applied Laser, Photonics and Surface Technologies, BFH, Bern University of Applied Sciences, Quellgasse 21, 2502 Biel/Bienne, Switzerland; § Laboratory for Mechanics of Materials and Nanostructures, Empa, Swiss Federal Laboratories for Materials Science and Technology, Feuerwerkerstrasse 39, 3602 Thun, Switzerland

**Keywords:** TOFMS, HiPIMS, IEDF, microwave plasma, in situ diagnostics

## Abstract

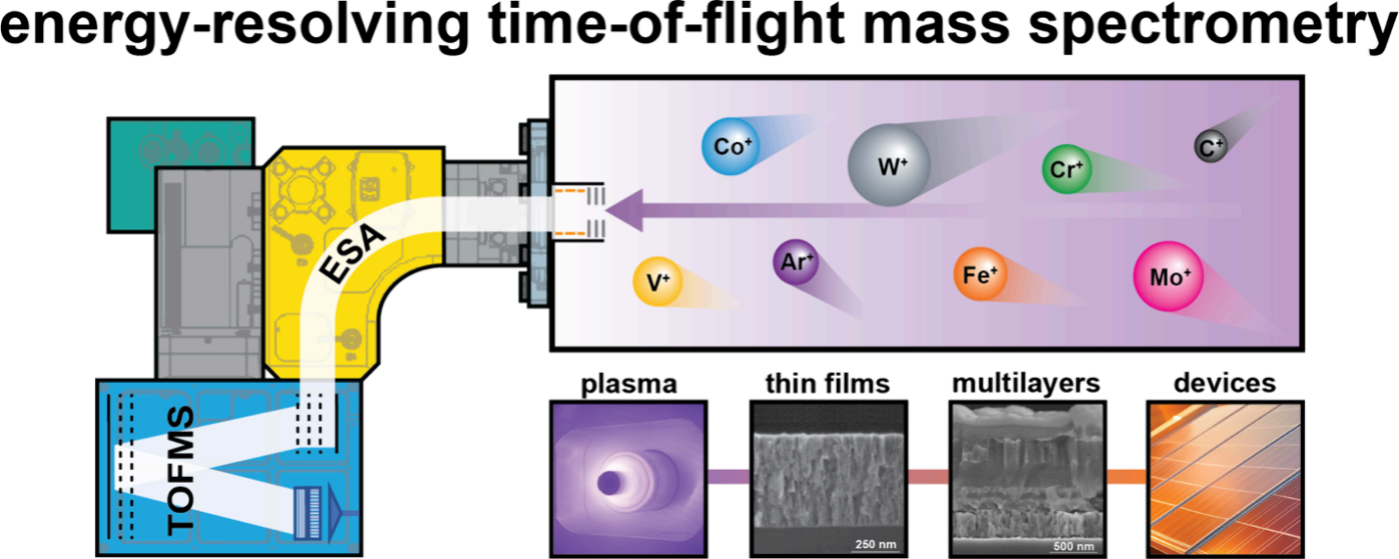

This work presents a newly designed energy-resolving time-of-flight mass spectrometer (E-TOFMS) for analysing the energy and mass of ions in bulk plasma. The system comprises an electrostatic sector analyser (ESA) for energy-to-charge (E/Q) ratio resolution and an orthogonal reflectron TOFMS for mass-to-charge (m/Q) ratio analysis. The design choices are explained, providing insight into electron and ion path simulations. The instrument was characterised using various ion generation sources, including an electron impact ion source, high power impulse magnetron sputtering, and microwave plasma electron cyclotron resonance sources. To validate its functionality, the energy-resolving data was compared with data obtained under the same conditions using a Langmuir probe and a retarding field energy analyser (RFEA). The benefits of the proposed E-TOFMS were demonstrated by sputtering highly alloyed steel with multiple isotope-rich elements, such as Mo or W. This technique offers an E/Q ratio resolution of up to 0.15 V for a range up to 125 V and a m/Q ratio resolution of at least 700 Th for a range up to 250 Th, with a temporal resolution of 10 μs.

## 1 Introduction

The field of materials science, and in particular thin film deposition processes, has undergone significant developments that go beyond the mere control of chemical composition and into the realm of control of the deposition environment. The work of J. A. Thornton on the structure zone diagram (SZD),^1^ as well as A. Anders’ version extended to include plasma-based phenomena,^2^ clearly illustrates the importance of process parameters and their influence on the properties of the deposited film. In the extended version, A. Anders replaced the pressure axis with the reduced kinetic energy of the impinging species, whereas the reduced temperature axis was modified to account for the potential energy of the species. Several studies have demonstrated the influence of energetic species on a range of processes, including ion bombardment-induced stress in AlN films^3^ and the role of ion energy in the formation of sp^3^ rather than sp^2^ bonds for the growth of hard diamond-like carbon (DLC) films during magnetron sputtering.^4^ In the latter case, both the species energy and the species itself are of importance, where energetic C^+^ favours sp^3^ bonds via the subplantation mechanism, while energetic Ar^+^ bombardment results in softer sp^2^-rich films. The ability to estimate the energies of ionic species can also facilitate the linking of microstructure and crystallographic structure changes of thin film materials when varying the deposition environment conditions, e.g. comparing direct current magnetron sputtering (DCMS) with high power impulse magnetron sputtering (HiPIMS), investigating the influence of substrate bias and/or testing different plasma source combinations.^4−7^ These effects are not limited to single layers but can also affect the interface between subsequent layers in a multilayer structure, such as in photovoltaic (PV) or passive daytime radiative cooling (PDRC) devices,^8−12^ where the sequence, thickness, composition, structure and properties of each individual layer, as well as the quality of the interface, are critical to the proper functioning of the device.^6,7,13^ Therefore, a comprehensive understanding of the deposition conditions is necessary for the controlled and well-defined manufacturing of modern materials and devices.

Photon-based methods, such as optical emission spectroscopy (OES), can provide valuable information on the deposition environment.^5,13,14^ For instance, Thomson scattering can determine electron density and energy distribution,^15,16^ while tunable diode laser-induced fluorescence (TD-LIF) can measure the velocity distributions of atoms and ions.^17^ Although these techniques can offer valuable insights, they are often too demanding in the context of thin film development. Retarding field energy analysers (RFEA) can be used to directly measure the ion energy distribution function (IEDF),^18,19^ and when combined with a quartz crystal microbalance, they can also determine the ion and neutral flux.^13,19^ However, these techniques do not provide information on the nature of the flux. Several mass spectrometric techniques have been employed to quantify the ion flux generated in a plasma.^19^ In the field of chemical analysis, magnetic sector inductively coupled plasma (ICP) and time-of-flight mass spectrometry (TOFMS) systems are commonly used due to their high resolving power.^20,21^ The characterisation of plasma-based PVD processes has been conducted using energy-resolving quadrupole mass spectrometry (QMS), with the initial publications dating back to the 1970s. The approach, as described by Coburn,^22,23^ involved the use of a spherical electrostatic energy analyser (ESA) in combination with a QMS. Since that time, numerous studies have employed this technique for the diagnosis of plasmas with simple chemistry, where the limited resolving power of QMS systems (1 Th) does not cause significant drawbacks.^24−26^ Quadrupole systems operate sequentially to acquire mass spectra and can have a duty cycle close to 100 % for a single mass-to-charge (m/Q) setting. However, when multiple elements and isotopes need to be detected consecutively, the duty cycle is divided by the number of m/Q ratios of interest, which significantly reduces the duty cycle in the context of complex applications, such as (co-) sputtering of multiple and/or alloyed targets. In comparison, TOFMS has the intrinsic ability to measure m/Q ratios quasi-simultaneously over a large mass range, with significantly higher mass resolution.^27^

This work presents an energy-resolved TOFMS (E-TOFMS) that combines well-established TOFMS with an ESA, to allow for simultaneous identification of plasma ion species (m/Q range deliberately limited to 250 Th, however, can be increased up to 6000 Th) including their energy-to-charge (E/Q) ratio. Other ion energy filter design concepts, such as mirror-type and deflector plate analysers, have been extensively discussed in literature^28^ and used in combination with QMS^29^ and TOFMS^30^ systems. ESAs use radial or spherical electric fields to bend and partially focus ion beams. The cylindrical deflector plate analysers, introduced by Hughes and Rojansky in 1929^31,32^ and commonly used,^28,33−37^ focus the ion beam only in the dispersion direction. Matsuda in 1961^38^ proposed adding two additional electrodes to achieve ion beam focusing in both directions. This energy band-pass filter design ensures that the ions fed to the TOFMS’s entrance exhibit a narrow energy-to-charge ratio band, enabling quantitative and high-resolution mass spectrometry.^39^ The energy-to-charge ratio of the ESA can be adjusted to meet specific requirements for energy resolution, transmission efficiency, and maximum ion energy. Instruments commonly used for plasma diagnosis in the context of thin-film deposition, employ a 45° cylindrical ESA, focusing the beam in only one direction.^40−42^

This work aims to explain the design and operation of the new E-TOFMS and demonstrate its capabilities for *in situ* characterisation a wide range of deposition environments. To validate the measured results, they are compared with other state-of-the-art plasma analytical devices, such as Langmuir probe and RFEA measurements.

## 2 Experimental Description

### 2.1 Chamber Configuration

The deposition chamber used for all the presented case studies was a HEXL Modular Deposition System, equipped with two magnetrons in unbalanced configuration (Korvus Technology, UK). Argon was introduced at a flow rate of approximately 60 sccm, which resulted in a pressure of 0.48 Pa. Two HiPSTER 1 power supply units (Ionautics, Sweden) were used to initiate pulsed sputtering, and 3 Aura-Wave coaxial ECR plasma sources (SAIREM, France) enabled generating a microwave plasma filling the entire volume of the deposition chamber.^43,44^ Further details on the used deposition setup have been reported earlier.^4^

### 2.2 Diagnosis Equipment and Material Characterisation

Dedicated experiments were performed to validate the accuracy and resolving power of our ion energy-to-charge ratio (E/Q) measurements. The experiments used a crossbeam ion source that provides near monoenergetic ions at selectable ion energies. A commercial crossbeam electron impact (EI) ionisation source (Pfeiffer Vacuum GmbH) was mounted directly on the sample tube. Electrons emitted from a heated filament are accelerated into an ionisation chamber, where they ionise the residual gas (see Supporting Information Figure S1). The voltage of the ionisation chamber can be adjusted to vary its potential. The ions can then be extracted into the sample tube through an extraction orifice, gaining kinetic energy proportional to the potential difference between the ionisation chamber and the sample tube. Ion optics simulations were conducted to calculate the expected ion energy distribution and compared with the measured E/Q distributions. SF_6_ was added to the ionisation chamber through a leak valve resulting in a pressure range of 10^–4^ to 10^–3^ Pa. In addition to the SF_6_ peaks (127 Th for SF_5_^+^, 108 Th for SF_4_^+^ and, 89 Th for SF_3_^+^), air background peaks of H_2_O^+^ at 18 Th and N_2_^+^ at 28 Th were analysed for calibration.

Langmuir probe measurements were performed to determine the plasma potential, which gave an indication of the average ion energy. It also provided a measure of the plasma density in the vicinity of the orifice to estimate the likelihood of artefacts. In this context, a single Langmuir probe (Impedans Ltd., Ireland) was operated under sputter and microwave plasma conditions. The Langmuir probe consists of a 6 mm diameter anodised aluminium shaft with a 10 mm long tungsten wire tip attached to the end. The shaft is shielded by an additional grounded aluminium tube. A voltage sweep was performed from −20 to 30 V in 0.5 V increments and the resulting current was recorded.

Ion flux measurements were conducted with a Semion pDC System Standard Density probe (Impedans Ltd., Ireland). The system includes a retarding field energy analyser (RFEA) with three grids, which measures ion energies and ionic flux densities. The principle and description of this system have already been reported in previous works.^18^

The chemical composition of the sputter targets used was determined via X-ray fluorescence (XRF), using an X-ray XDV-SDD Fischerscope (Helmut Fischer AG, Germany). The beam energy was set to 50 kV, with a spot size of 0.3 mm.

### 2.3 E-TOFMS Configuration

The E-TOFMS was connected to the reactor through a DN100 ISO-K flange, with the distance between the orifice and the reactor’s flange being 95 mm. The sampling tube had an outer diameter of 64 mm. The E-TOFMS system, i.e. from the orifice to the TOFMS, was evacuated using a split flow turbomolecular pump (Pfeiffer SplitFlow 80) with a pumping speed of 60 L/s at the first (TOFMS) stage. The fore pump used in this study was an MD 1C VARIO-SP (VACUUBRAND GMBH + CO KG, Germany). The pressure in the sampling tube was maintained at 5 × 10^–3^ Pa. The residual gas pressure in the ESA was approximately 3 × 10^–4^ Pa, and in the TOFMS 10^–4^ Pa.

**Figure 1:**
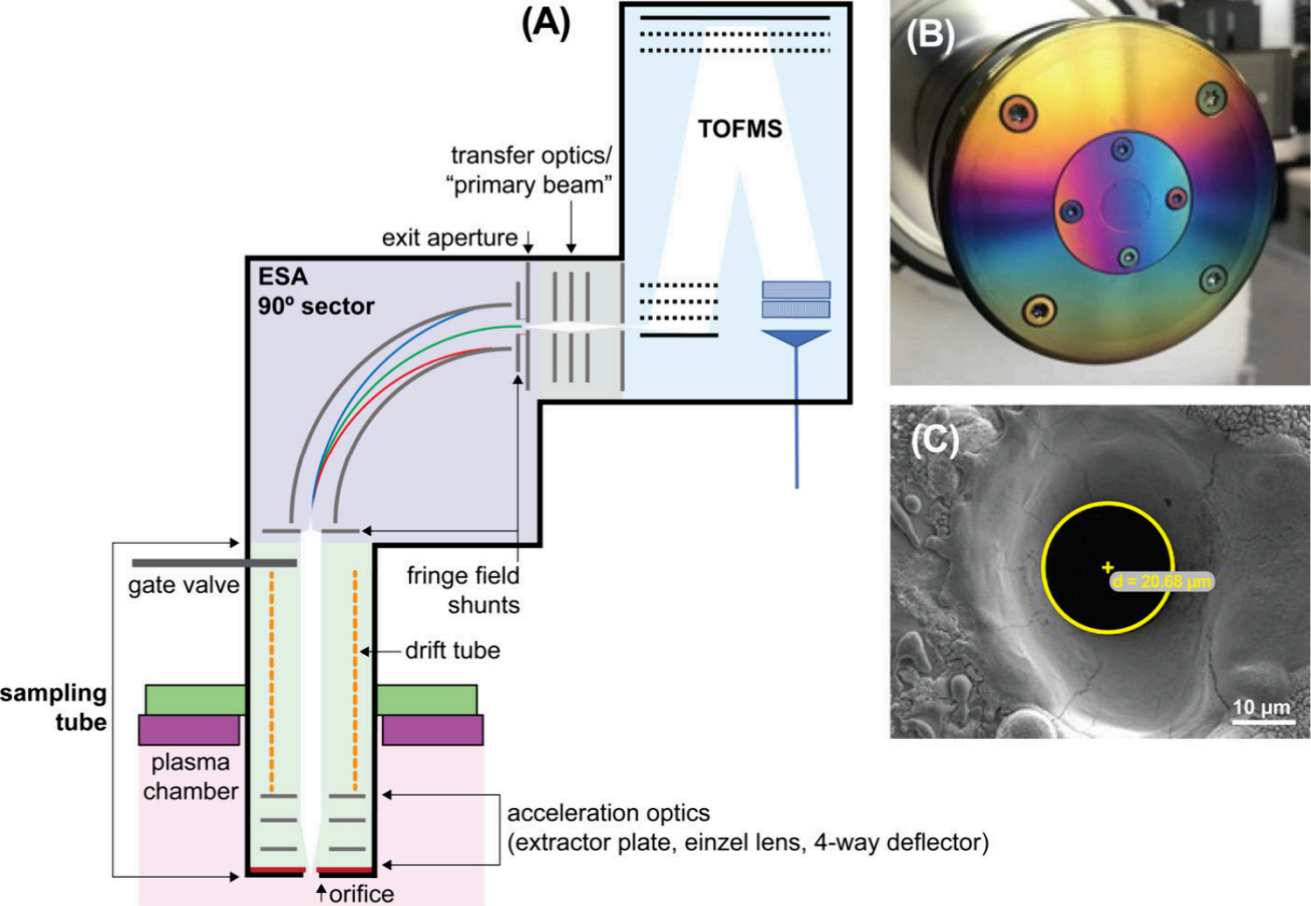
(A) Schematic of ion optics configuration of the E-TOFMS, (B) mounted orifice, (C) SEM image of the 20 μm diameter orifice aperture.

The E-TOFMS **ion optics** configuration is shown in Figure 1A. The ions travel from the plasma chamber to the **sampling tube** through an **orifice** (Figure 1B,C). The orifice, which can be biased, grounded, or kept on floating potential, is an optical aperture disc (SS-3/8-DISC, Lenox Laser) and consists of a circular aperture (diameters of 30 μm, 20 μm, 10 μm were used in this work) in a 50 μm thick stainless-steel foil. After leaving the orifice, the ions reach the **acceleration optics**. The extraction voltage can be scanned between 0 and 125 V to allow ions of different E/Q values to reach the E/Q set for the ESA. After the 7 mm acceleration stage, the ion beam is collimated with an **einzel lens**. This maximises acceptance as the lens is close to the orifice, and aberrations are reduced by using an asymmetric configuration of the einzel lens. A 4-**way deflector** is used to align the beam with the ESA. The ions are then guided to the entrance of the ESA using a 141 mm **drift tube**. A gate valve is incorporated to separate the ESA and TOFMS from the plasma chamber. Next, the ion beam enters the **90° ESA**, through a fringe field shunt in the Herzog configuration.^45^ The 90° ESA sector is comprised of two cylindrical electrodes with a mean radius of r_0_ = 100 mm. The distance between the inner and outer cylinder is 25 mm, and the height of the cylinder electrodes is 60 mm. Matsuda plates^38^ are placed at the bottom and top of the cylinder electrodes to create an approximately spherical electrostatic field near the optical axis. The spherical field leads to a higher energy-to-charge ratio separation and increased transmission due to two-dimensional focusing at 90°. The E/Q of the ions that pass the ESA on its optical axis is determined by the geometry and the potentials of the cylindrical electrodes and can be calculated using the following eq 1:
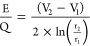
1where: (V_2_ - V_1_) is the potential difference between the outer and inner electrode, and r_2_ and r_1_ are the respective cylinder radii. During an energy scan, the passband E/Q of the ESA is fixed, and the acceleration potential is scanned. Ions with an initial E/Q equal to the difference between the pass energy and the acceleration voltage meet the pass condition. The voltage steps and durations during a scan can be selected as needed. The linear E/Q dispersion coefficient (D_k_) of an ideal 90° spherical deflector plate analyser is equal to the radius of the optical path corresponding to r_0_, therefore the E/Q resolving power (R_k_) using an exit slit of width S is given by eq 2:
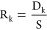
2

The R_k_ describes relative resolving power compared to the set E/Q of the ESA. The ESA’s narrow exit slit, which is adjustable (here set at 1 mm or 0.5 mm), is created by a second fringe field shunt. This shunt was designed using elements suggested by Jost^46^ and Pomozov and Yarov.^47^ The theoretical E/Q resolving powers with these shunts are 100 and 200, respectively. However, the experimentally determined resolving power may be reduced by various factors, such as higher order aberrations, imperfect ion beam alignment, and possible secondary electron emission.^28^ Additionally, the Matsuda plate configuration only mimics the spherical field, leading to an increase in higher order aberration coefficients. The ions proceed to the 32 mm long transfer optics, where they are either decelerated or accelerated to approximately 55 V. Lastly, they are collimated once again using two einzel lenses before reaching the entrance slit of the TOFMS.

The **mass spectrometer** used in this study is an orthogonal accelerating reflectron time-of-flight mass spectrometer (CTOF, TOFWERK, Switzerland). Data is acquired using a 14-bit, 1.6 GSPS analog-to-digital converter (ADQ1600, SP Devices). The time resolution of the system is limited by the flight time of the ions in the TOFMS and, therefore, depends on the highest mass-to-charge (m/Q) ratio that needs to be measured. The m/Q range covered is between 1 and 250 Th, with an extraction period of 10 μs. The dynamic range is 10^5^ for a 1 s acquisition (further details are provided in references^48,49^). The m/Q ratio resolving power (using the full width at half-maximum, FWHM) is 700 for low mass-to-charge ratios (^12^C^+^), 1200 for average mass-to-charge ratios (^56^Fe^+^), and 1600 for higher mass-to-charge ratios (^184^W^+^). This mass resolution is sufficient to identify multiple charged ions with a noninteger m/Q, such as Al^2+^ and Ar^3+^, and is adequate to distinguish between typical carbohydrates and metal ions, e.g. C_4_H_9_^+^ and ^55^ Mn^+^. However, it reaches its identification limit for ^44^Ca^+^ and CO_2_^+^, and is unable to distinguish between ArH^+^ and AlN^+^.^42^

The duty cycle of the orthogonal TOFMS extraction is determined by the ratio of the time required for the ion flux to pass through the extraction tube of length L to the TOFMS extraction period T_ext_ (eq 3). In this configuration, ions enter the extractor with a defined energy-to-charge ratio (E/Q)_ext_. Accounting for the m/Q, the mass-dependent duty cycle becomes straightforward and reliable^50^ (see Supporting Information Figure S3).
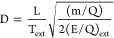
3

For an extraction time of 10 μs, extraction tube length L of 46 mm and an extraction energy-to-charge ratio (E/Q)_ext_ of 55 V, the duty cycle varies from 16 % for ^12^C^+^ to 60 % for ^184^W^+^. This means that the ion counts measured for a single extraction correspond to a time integral of 1.6 μs for ^12^C^+^ and 6 μs for the heavier ^184^W^+^ single charged ion. As mentioned in the introduction, if multiple elements are to be detected by a QMS, this must be done sequentially for each m/Q value, which significantly reduces the duty cycle (e.g., for a single m/Q measurement, the duty cycle would be close to 100 %, whereas if ten elements are to be measured, the duty cycle would drop to 10 %).

The ion transmission through the aperture depends on its size. It is therefore difficult to estimate the angle dependent transmission of the orifice. Using a 50 μm thick orifice plate with a 20 μm diameter hole, the geometric half angle is . However, other factors, such as hydrodynamic gas flow through the orifice or electrostatic interactions of ions with the orifice wall can also affect the transport of charged particles and increase the measurement uncertainty.

The pressure inside the sampling tube and after the orifice is less than 5 × 10^–3^ Pa and the mean free path of the ions is much longer than the length of the sampling tube. Therefore, interactions of the ions with residual gas and wall effects can be neglected.

The ion transmission through the E-TOFMS was simulated with SIMION (version 2021–06–26–8.2.0.11). The ion transmission from the orifice to the extractor of the TOFMS for ions originating at the orifice exit depends on their E/Q and their entrance angle. For ions with small angles or low E/Q values, a transmission close to 100 % is expected. As the E/Q increases, the transmission decreases for ions with larger angles. For an ion with an E/Q of 20 V, 90 % transmission is achieved at an angle of 12.5°, while 50 % transmission is observed at 17.5° for an E/Q of 50 V (see Supporting Information Figure S2 for other angles and energy-to-charge ratios).

Since all ions in the ESA are accelerated to the same E/Q value and only electrostatic fields are used (no quadrupole RF fields), the time-of-flight of the ions through the energy analyser is determined by the E/Q value of the ESA and the m/Q of the ion and is almost independent of the ion energy. Only in the acceleration region, where the incoming ions are accelerated to the ESA energy, does the ion flight time depend on the incoming energy. However, the acceleration distance L= 7 mm makes up only a small portion of the total flight path, from orifice to extractor. The ion flight time from the orifice to the TOFMS extraction region, as derived from SIMION simulations, is (eq 4):
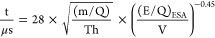
4where: m/Q is in Th and E/Q_ESA_ is the set passing E/Q of the ESA. Using this time lag, the measured E/Q and mass spectra can be precisely synchronised with the investigated plasma processes.

## 3 Results and Discussion

### 3.1 Energy-to-Charge Ratio Calibration

**Figure 2:**
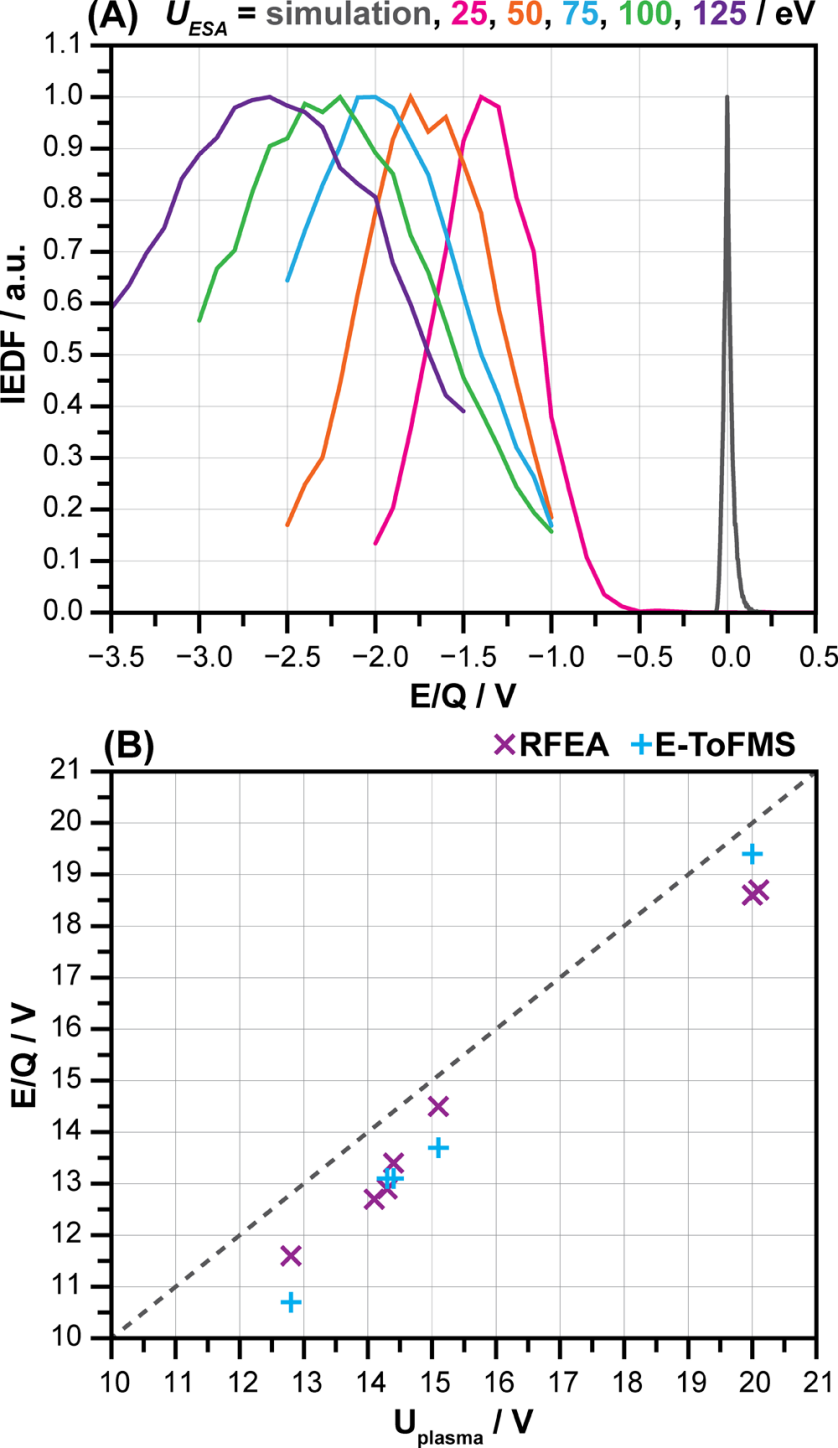
(A) Simulated vs measured IEDFs for different set E/Q of the ESA (E/Q_ESA_) measured using an EI ion source with optimised settings. (B) Peak value of IEDF measured via the RFEA and E-TOFMS vs the plasma potential U_plasma_ determined by the Langmuir probe (measuring conditions: microwave plasma generated by 3 Aura-wave applicators of varying power: 50 to 150 W, pressure: 0.47 and 0.6 Pa and Ar/N_2_ gas mixtures).

In principle, an ion E/Q can be calculated directly from the known geometry and voltages applied to the ESA electrodes without any further assumptions. However, small mechanical or electrical inaccuracies, such as machining tolerances leading to misalignment or electrical noise, could lead to deviations from the theoretically derived ion energies. Therefore, to further improve the accuracy of the E/Q data, measurements were made with ions from an EI source. To achieve this, it is necessary to operate the EI source with optimised settings. First, it is important to consider the space charge of the electrons inside the ionisation chamber. The higher the emission current of the filament, the greater the influence of the space charge on the electric field inside the ionisation chamber. A linear decrease in the measured ion energies of 0.1 eV per 0.1 mA increase in emission current was observed. Thus, the emission current was set to 0.1 mA and extrapolated to 0 mA by adding 0.1 eV to the measured data to correct for the space charge effect. Additionally, the electric field used to extract the ions from the ionisation chamber may affect the homogeneity of the electric field. For this reason, the extraction plate potential of the ion source (adjacent to the ionisation chamber) and the orifice plate potential were kept at the same level as the ionisation chamber. The ions were then extracted solely by the weak field from the extractor voltage inside the E-TOFMS sampling tube. Simulations indicate that under these conditions, the ion energy distribution of the extracted singly ionised ions is symmetrical, centred around the potential of the ionisation chamber and with a FWHM of approximately 0.1 eV. The expected ion energy was compared with the measured distribution for various ESA settings (10 to 125 V) and ionisation chamber voltages (0 to 100 V). Figure 2A shows that the measured ion energies are −1 eV to −2.5 eV lower than the known E/Q of the ions generated by the EI source. This offset linearly depends on the ESA’s energy (eq 5).
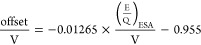
5

The E-TOFMS scan software takes this offset into account and reports calibrated ion energies. The measured peak ion energies of singly charged ions scale linearly with the ionisation chamber voltage of the EI source in the range of −10 V to +100 V (linear least-squares fit: y = 0.998 · x–0.16, R^2^ = 1).

To verify the accuracy of the E-TOFMS, a comparison was made between the IEDF of the RFEA sensor and the E-TOFMS by measuring a microwave plasma generated at different power settings, pressures and Ar/N_2_ gas mixtures. The plasma potential was measured by means of a Langmuir probe. Figure 2B shows that the peak energies of the IEDF determined by both methods are slightly below the plasma potential measured via a Langmuir probe (approximately 1 V) under the same conditions. The plasma potential, derived from the Langmuir probe measurements, and the maximum of the IEDF are related, but not identical. A small systematic shift can therefore be expected. The measurement uncertainties, visible in the scatter of the measurement results, are caused by a combination of different effects, including the uncertainty in the determination of the IEDF peak (0.1 V), the reproducibility of the IEDF measurement (0.5 V), which is influenced by, among other things, the mounting and dismounting of sensors and plasma sources, the purity of the carrier gas and the temperature.

**Figure 3:**
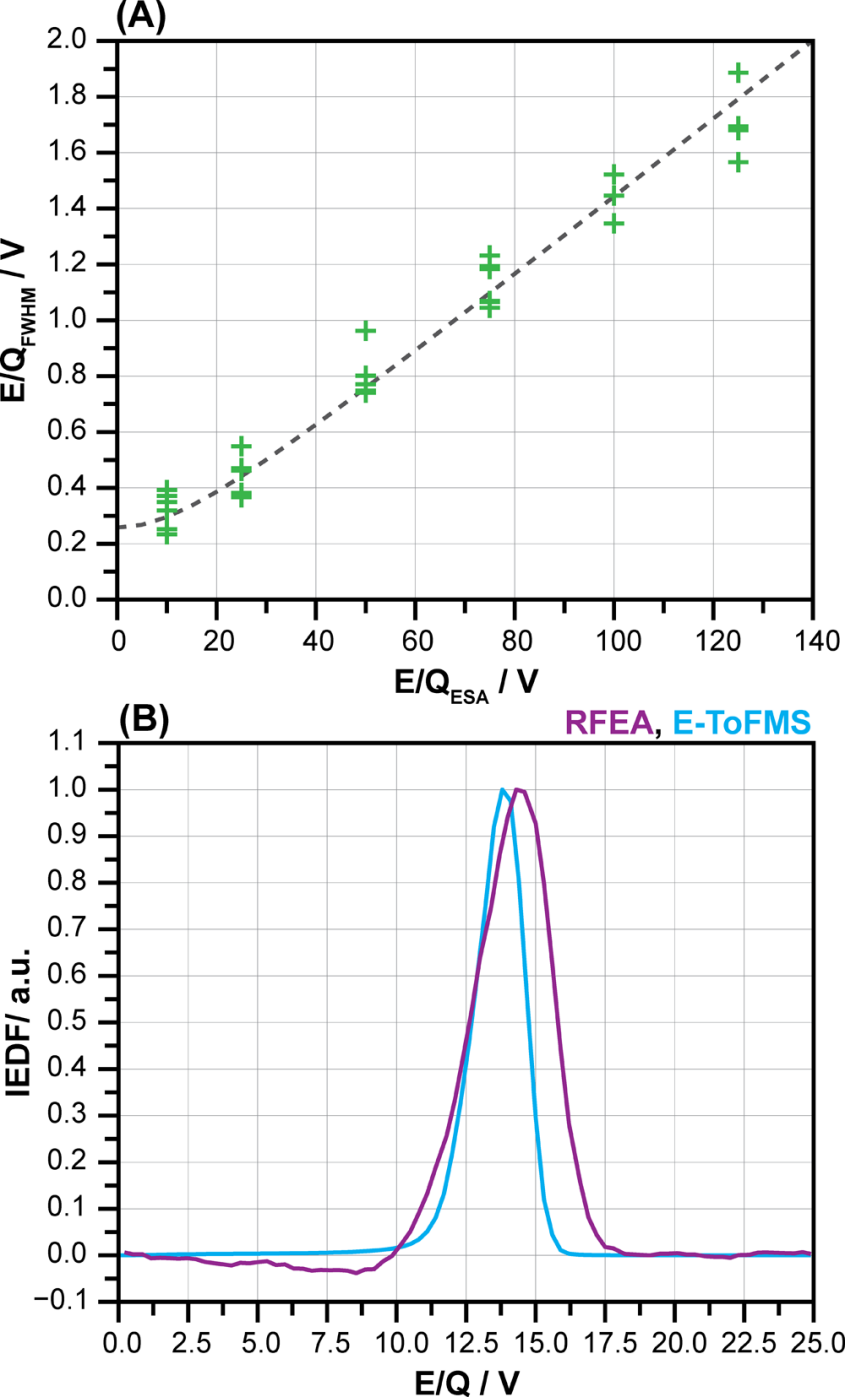
(A) The energy-to-charge resolution (E/Q_FWHM_) measured as a function of the ESA energy-to-charge ratio (E/Q_ESA_) using a 0.25 eV ion beam produced by an EI source for a 1 mm exit slit, (B) IEDF measured via an RFEA and the E-TOFMS for a 3 × 50 W power Ar microwave plasma, pressure 0.47 Pa, where the RFEA current derivative was calculated with a 0.6 V standard deviation Hann window and the E-TOFMS measured the intensity of the Ar^+^ peak at 40 Th.

The E/Q resolution was measured by scanning the ion E/Q of the ion beam produced by the EI source (for a 1 mm exit slit). Figure 3A displays the peak widths of the E/Q for different ESA energy-to-charge ratios. The E/Q_ESA_ points correspond to SF_6_ peaks (127 Th for SF_5_^+^) and air background peaks (H_2_O^+^ at 18 Th, N_2_^+^ at 28 Th) of the mass spectrum. Each peak width was measured twice. The initial E/Q of the ions had no systematic effect on the width of the E/Q peak. The measurement uncertainties, visible as scatter in Figure 3A, are caused by a combination of several effects including low ion counts, different m/Q and varying extraction voltage settings. The experimental data can be explained as the result of the convolution of two distributions. The ion beam’s energy distribution (0.25 eV width, as determined by SIMION simulation) and linear dispersion were used in the ESA. Based on this, the resolving power using a 1 mm exit slit is 71, which is approximately 30 % lower than the maximum resolving power achievable by an ideal 90° spherical sector analyser (R_K_ = 100). It is important to note that these measurements were taken with a voltage setup that maximised ion transmission. Using a different voltage setup that minimises beam emittance at the ESA entrance and maximises E/Q dispersion inside the ESA could result in higher resolving powers, however, at the expense of ion sensitivity. The width of the distributions analysed is significantly larger than the resolution of the instrumental E/Q. The exit slit size was adapted to the applications studied in this work, however, it can be adjusted to meet specific requirements. A 0.5 mm wide exit slit would approximately double the resolving power of the E/Q to about R_K_ ≈ 140. Therefore, for small ion energies with the ESA set to 10 eV, resolutions of <0.1 eV are expected to be achievable. High energy resolution can be important for determining the ion energy limit between atomic layer etching and physical sputtering^51^ and/or for determining the ion temperature in a plasma, as discussed by Cunge et al.^52^

A comparison was conducted between the IEDF measured using the E-TOFMS system and the commercially available RFEA sensor (Figure 3B). The same microwave plasma discharge conditions described previously were used to generate the ion population to be probed by both systems. The FWHM of the IEDF was found to be 2 eV using the E-TOFMS, which was independent of the ESA energy settings and larger than the instrumental contribution. In contrast, the 3-grid RFEA probe had a FWHM of 3 eV. In addition to the previously discussed causes of measurement uncertainty, the differences in IEDF measured by the two instruments are amplified by the contribution of secondary electrons to the RFEA probe, lensing effects at low energies and possible long-term fluctuations in microwave plasma generation (see Supporting Information Figures S4–7 for more details).

### 3.2 Example Applications

This section presents a selection of E-TOFMS measurements carried out in relation to high power impulse magnetron sputtering (HiPIMS) thin film deposition. The purpose of these examples is to evaluate and demonstrate the instrument’s performance in a typical thin film process environment.

**Figure 4:**
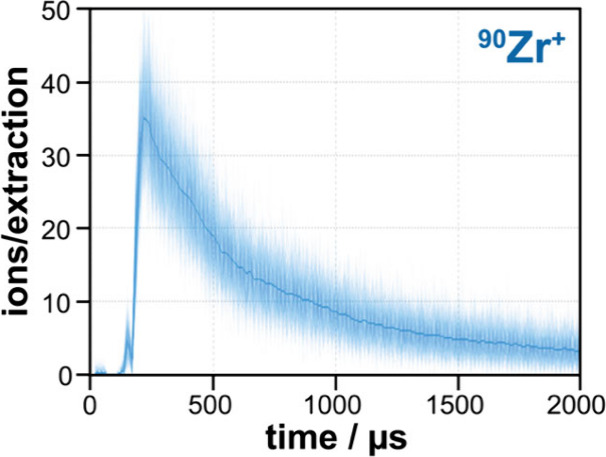
Single pulse analysis during zirconium HiPIMS, measurement initiated 20 μs before the start of the pulse.

The first example shows the **temporal ion flux variation during a single HiPIMS pulse**. A zirconium target was used in an argon atmosphere with a pressure of 0.6 Pa. The peak current was 5 A, and the pulse length was 100 μs with a repetition rate of 100 Hz. Figure 4 shows the ion counts for consecutive extractions at 100 kHz (i.e., 200 data points with 10 μs intervals) for a constant energy setting. The ion flux is highest immediately after the end of the HiPIMS pulse. The count rate, i.e. the ion population in the reactor, then slowly decreases as the ion density is reduced by diffusion and recombination. The extraction-to-extraction reproducibility follows a Poisson distribution, as expected in this case. This example illustrates the sensitivity of the E-TOFMS system in characterising transient events, such as the measurement of the ion spectrum produced by a single HiPIMS pulse affected by arcing. Furthermore, although only the behaviour of Zr during a single HiPIMS pulse is shown here, in fact all m/Q ratios were collected for the exact same pulse, which would not be possible using a sequential QMS system (see Supporting Information Figure S8 to see the behaviour or Ar^+^ during the same pulse).

**Figure 5:**
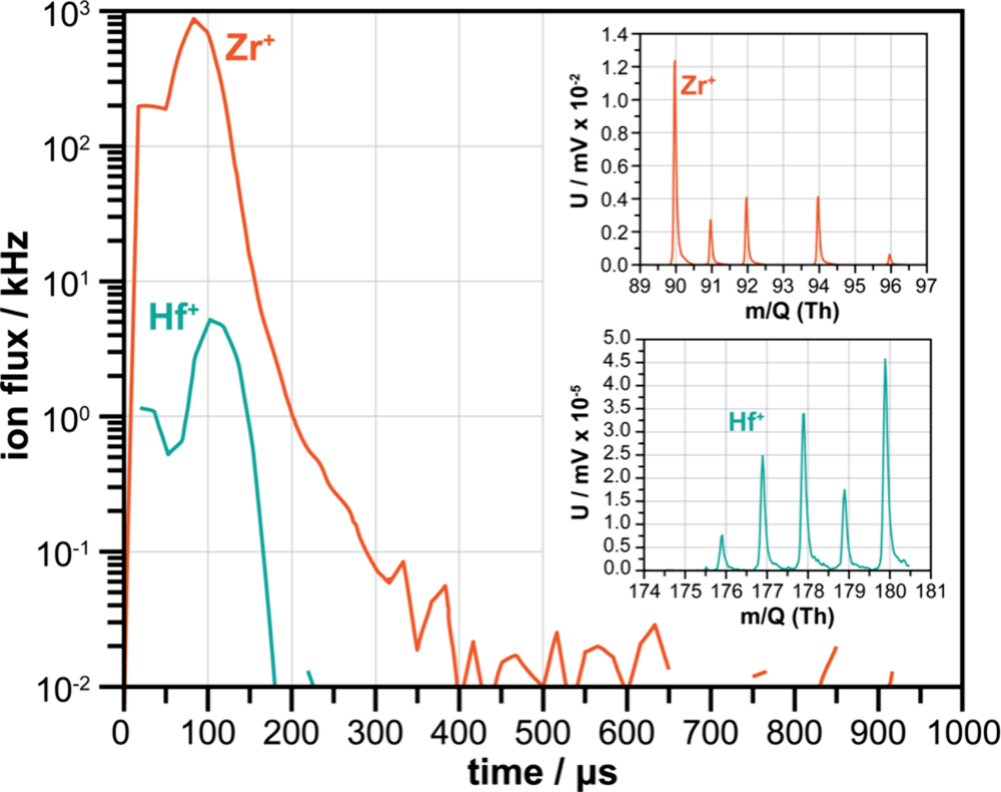
Energy-averaged pulse profile for Zr^+^ and Hf^+^, summed over all isotopes, for a 50 μs pulse width HiPMS discharge for a Zr target, with inserts showing the mass spectra for Zr^+^ and Hf^+^ isotopes (U corrected for the duty cycle and scales adapted to data range).

A different application example concerns the **mass independence of ion flux measurements**, independent of the energy-resolving capability of the E-TOFMS. Although calibrated ion beams would have been ideal for the experimental assessment of the mass dependent ion transmission, they were not available. Instead, the E-TOFMS system was tested by characterising the ion flux produced by sputtering from a 2″ Zr target (with a Hf content of 1 at%, as measured by XRF) via HiPIMS. Due to the lanthanide contraction, Zr and Hf have very similar valence electron configurations. Their atomic radii are approximately the same, and their first ionisation energies are 6.34 and 6.83 eV, respectively. Their electronegativity values are also similar, with Zr at 1.33 and Hf at 1.30.^53,54^ Therefore, it can be assumed that their ionisation cross-section in an Ar HiPIMS discharge is comparable. Figure 5 displays the mass spectra and time-resolved ion fluxes measured simultaneously for Zr and Hf in energy-averaged mode. The surface area of the m/Q reproduces the natural isotopic abundance of the two elements. The duty cycle-corrected ion fluxes (see eq 3) show an Hf^+^ ion abundance ratio of 0.7 % of detected metal ions, slightly below the atomic Hf concentration of 1 at% measured by XRF. The difference between the measured ion flux and elemental concentration can be attributed to various factors, including the HiPIMS plasma, mass dependent ion transport from the plasma to the substrate area, and the velocity dependence of the single ion signal produced at the detector. To reduce the effect of the latter, a mass dependent calibration of the single ion signal can be employed. The other effects provide information on the deposition process.

The following example focuses on demonstrating the ability to characterise the **temporal and energetic distribution of complex deposition environments**, such as when sputtering from a highly alloyed 2″ target made of high speed steel S290 (Böhler) via HiPIMS. Table 1 summarises the chemical composition of the target, which contains both light (e.g., carbon) and heavy (e.g., tungsten) elements, as well as the relative sputtered ion fluxes of each element obtained via E-TOFMS. The HiPIMS conditions used were: 200 Hz sputtering frequency, 50 μs pulse width, and 17 A peak current, while the E-TOFMS settings were: E/Q_ESA_ of 50 V and exit slit size of 0.5 mm. The HiPIMS pulse was delayed by 20 μs relative to the start of the E-TOFMS trigger.

**Table 1:** Chemical Composition of the S290 High Speed Steel Measured via XRF and Energy- and Time-Integrated Relative Ion Flux Fractions as Measured via E-TOFMS

element	Fe	C	V	Cr	Co	Mo	W
**steel chemical composition/at%**	62	10	5.1	4.5	10	1.5	6
**total ion flux fraction/%**	70.2	0.5	9.4	6.1	10.9	1.4	1.5
**fraction of doubly charged ions/%**	3.7	0.3	4.8	5.4	1.8	6.2	5.3

**Figure 6:**
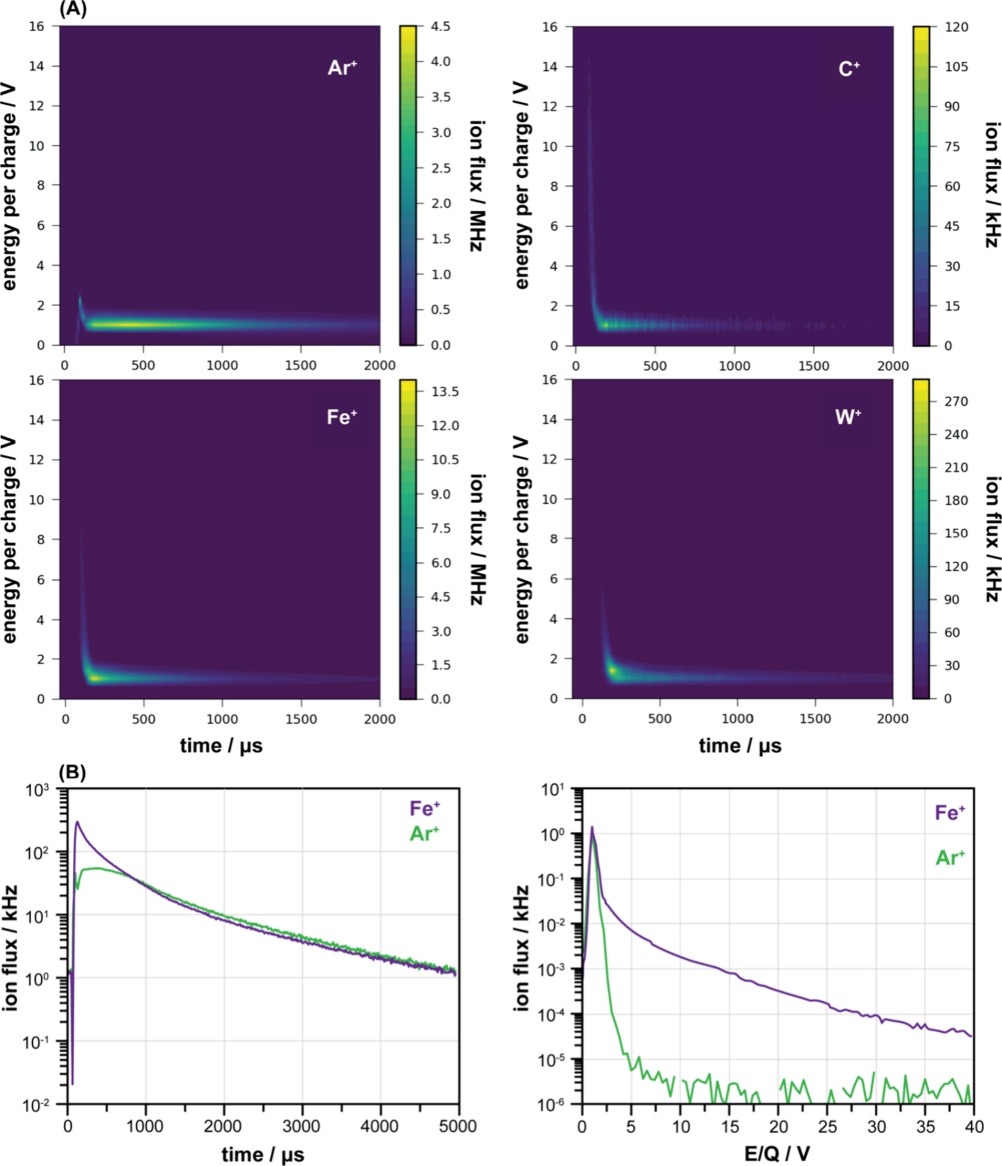
(A) E/Q distribution change during pulse for different species, (B) E/Q-averaged and time-averaged profile for the two most common species.

Figure 6A displays the temporal evolution of the C, Fe, W and Ar ion fluxes (the behaviour of Fe is representative for V, Cr, Co and Mo), and Figure 6B illustrates the difference in temporal changes between the ion energy distribution of Fe^+^ and Ar^+^. Shortly after the HiPIMS pulse ends, a strong maximum is detected for most of the measured ions. Ions related to sputtered materials, in contrast carrier gas related ions, exhibit a wide E/Q distribution (Figure 6B). After the initial influx of high energy ions has passed, both carrier gas ions and sputtered ions exhibit a slow exponential decay, characterised by a narrow energy E/Q distribution of low (1 V) peak value (see1 V) peak value (see Supporting Information Figure S9 for example mass spectrum).

Although all constituents of the target material, including carbon, produce a corresponding flux of singly and doubly charged ions at the orifice, there are noticeable differences in their relative importance, as shown in Table 1. The E-TOFMS technique measures different masses simultaneously while scanning the ion energy-to-charge ratio. Drifts in the sputter process, caused by target heating, pressure variations, or preferential sputtering, should not affect the relative abundance of the measured ion fluxes. The reasons for the observed differences were not investigated in this work. However, the low flux of carbon ions is likely due to the low ionisation rate of carbon in an argon plasma, which is caused by its relatively high first ionisation potential of 10.2 eV. The distribution of sputtered material leaving the target area depends on its mass. The ionisation yields of atoms in a plasma are influenced by various parameters, such as ionisation potential, plasma density, electron energy distribution function, and the spatial overlap of sputtered materials with energetic electrons. In the context of film deposition and control over the structure, microstructure and/or applicational properties of the fabricated film, then possessing the above-mentioned information would enable, among other things, to set up bias/pulsed bias schemes, e.g. changing the bias voltage to modify the energy of incoming ions positive pulses to reject high energy ions having a broad distribution, followed by a negative pulse, to have a narrow, well-defined energy distribution of ions involved in film growth.^6,26,55^ This is particularly important when considering that the energy associated with point defects is typically in the range of 1 eV, and therefore the energies of the impinging particles achievable in plasma-based vacuum processes ranging from 1 to 100 eV can strongly influence the fabricated material. Furthermore, it is important to be able to detect the presence of not only singly charged ions, but also doubly charged ions, as their kinetic energy will be doubled (for the same potential drop). Finally, in addition to kinetic energy, the impinging ions are also carriers of potential energy, which, as mentioned in the introduction when describing the SZD^2^, can have a significant impact and should not be neglected.

## 4 Conclusions and Outlook

This work presents the design and *in situ* analytical possibilities of a novel energy-resolved time-of-flight mass spectrometer in the context of thin film deposition processes. Unlike sequential QMS, the duty cycle of TOF systems that simultaneously measure the full mass spectrum is independent of the number of m/Q values measured. The E/Q ratio can be scanned in variable steps from 0 to 125 V, allowing the E/Q ratio resolution (0.1 V - 1 eV) to be adapted to the application requirements. The E/Q ratio resolution of 0.15 V is significantly lower than retarding field systems^30^ and comparable to various cylindrical ESA systems described in the literature. The E/Q result is sufficient for thin film deposition applications where the widths of the ion energy distribution functions are in the single eV range or larger. The sensitivity of the E-TOFMS was sufficient for all measurement conditions, including the analysis of single plasma pulses of multiple ions, which is not possible with a sequential QMS system. The instrument offers further optimisation possibilities. The orifice diameter can be easily adjusted to change the measurable ion flux range. Pulse analysis time resolution can reach up to 10 μs for a m/Q range of 250 Th, which can be extended to 6000 Th by reducing the time resolution. For the example applications, the settings for acquiring a m/Q range up to 200 Th were generally used. The mass resolution of 700–1600 is significantly higher than that of QMS systems and allows the distinction of multicharged ions with noninteger m/Q values, such as Al^2+^ Ar^2+^, from single-charged ions such as CH^+^ and N^+^. It is worth noting that there is a simple way to significantly improve the performance of this new instrument without requiring major design changes. One option is to replace the CTOF analyser used in this study with an HTOF or LTOF (TOFWERK AG, Switzerland), which would offer mass resolving powers of at least 3000 and 6000, respectively,^56^ which, for example, would allow for distinguishing ArH^+^ from AlN^+^.

Overall, E-TOFMS is a versatile tool for the analysis of complex plasma processes involving multiple energetic and chemical species, therefore in particular suitable for supporting development in thin film technology, where the IEDF for multiple ions is of interest. Examples of such areas include the development of high entropy alloy or compound films, where the energy of the impinging species influences the structure and microstructure of the grown film,^6,13,57,58^ or optimisation of thin film systems and interfaces in electronics, photovoltaics, and energy storage. The presented applications focus on PVD, specifically magnetron sputtering, however, it is likely that it will prove to be useful in the context of plasma enhanced chemical vapor deposition and atomic layer deposition (PE-CVD, PE-ALD)^27^ in general, where information on the IEDF and the nature of the ionic species involved in film growth are important for understanding the growth mechanism. Such measurements using conventional energy-resolving QMS systems would be greatly hindered, as it is not possible to measure more than a single m/Q ratio at a time, making it more difficult to follow ongoing plasma discharge processes and/or identify and characterise process instabilities, with the E-TOFMS additionally offering improved m/Q and E/Q resolution.

The E-TOFMS presented in this work can also be applied in the field of propulsion thrusters,^30^ where determining the energy distribution of ions in the propulsion plume is of utmost importance. The high mass resolution of the orthogonal TOFMS using a reflectron provides an advantage.^28,59^

To develop this instrument further, the integration of the negative ion mode and an additional ionisation source for the measurement of neutrals in addition to the native ions will be considered. By combining the instrument with a RFEA, absolute species resolved ion fluxes can be measured, which is not possible with currently available instrumentation.

## Supporting Information Available

Contains further details related to the calibration and validation of the E-TOFMS instrument e.g. simulations vs experimental results, example mass spectra (PDF)
