# Supplementary material for: Energy-Resolving Time-of-Flight Mass Spectrometry for Bulk Plasma Analysis
Source: J Am Soc Mass Spectrom. 2024 Jul 11;35(8):1786–96. doi: 10.1021/jasms.4c00140 (PMC11311225; doi:10.1021/jasms.4c00140)
Supplement: Supplementary file 1 — js4c00140_si_001.pdf [file js4c00140_si_001.pdf]

SUPPORTING INFORMATION

Energy-Resolving Time-of-Flight Mass Spectrometry for Bulk Plasma Analysis

Malte Watzek [a,b], Patrick Sturm [a], Carsten Stoermer [a], Abdelhak Bensaoula [a], Thomas Nelis [b,c], Caroline Hain* [b,c]

[a] TOFWERK AG, Schorenstrasse 39, 3645 Thun, Switzerland
[b] Institute for Applied Laser, Photonics and Surface Technologies, BFH, Bern University of Applied Sciences, Quellgasse 21, 2502 Biel/Bienne, Switzerland
[c] Laboratory for Mechanics of Materials and Nanostructures, Empa, Swiss Federal Laboratories for Materials Science and Technology, Feuerwerkerstrasse 39, 3602 Thun, Switzerland

*corresponding author: e-mail: caroline.hain@empa.ch

1. EI ionisation source simulations and measurements

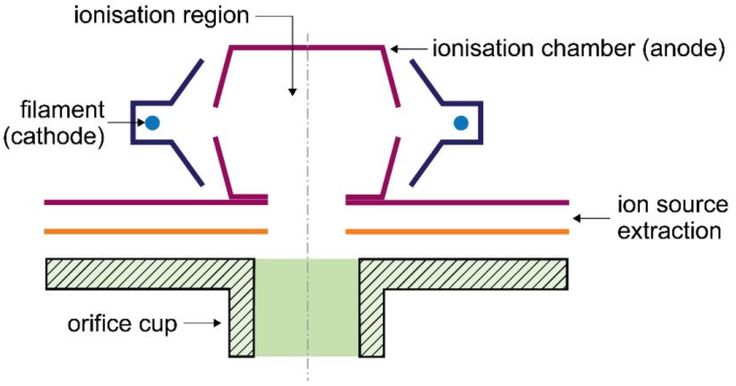

Figure S1 Schematic of the EI ionisation source;

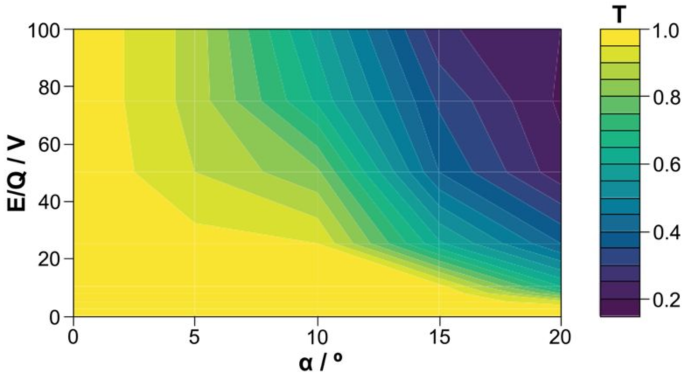

Figure S2 Simulated ion transmission *T* of the ion optics for different angles to the optical axis *α* and energy-to-charge ratios E/Q;

Figure S3 shows the integration time t_i as a function of m/Q for the setup used and an E/Q of 50 eV. The duty cycle D (Eq. 3 of the main article) is equal to the integration divided by the extraction pulse interval, in this case 10 µs. This means that with respect to the extraction pulse

event, ions entering the sample tube during t_i before extraction are considered. For a C^{2+} ion, this corresponds to a time of less than 1 µs, whereas for W^+ it is approximately 6 µs.

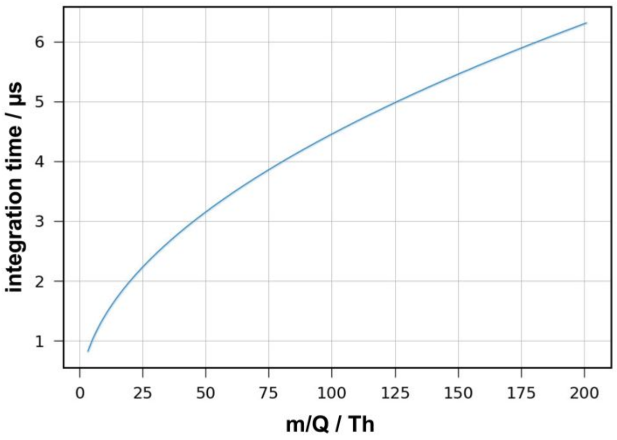

Figure 3 Integration time as a function of mass-to-charge ratios for an E/Q of 50 eV;

The average E/Q of ions passing through the energy filter was compared with simulated values using the EI source. The SIMION® software was used to simulate the electric fields and ion paths. The simulation of the ion source was performed in two steps: *(i)* simulation of the electron behaviour to determine the ionisation locations, and *(ii)* simulation of the ion paths to determine their E/Q upon arrival at the orifice. The electrons were randomly generated with a thermal velocity distribution at the filament surface. The ion trajectory simulations were followed by an ionisation simulation after a random distance (linearly distributed between 0 and 30 mm). At the collision point, an ion with a random thermal velocity distribution was placed. The ions were then simulated further, and the collision point with the electron was recorded for electrons reaching the orifice. This process reduced the number of collision points to the most useful. In the second step, these collision points were used as starting points for the ions. The path of the ions was simulated, and their kinetic energy was registered as they reached the orifice. The registered kinetic energies were then used to calculate the simulated IEDF. To obtain the narrowest possible energy distribution, different ion source settings were simulated. Figure S4 shows that reducing the number of collision points does not affect the resulting energy distribution, which is very important for useful results. The simulation time was reduced by a factor of ten.

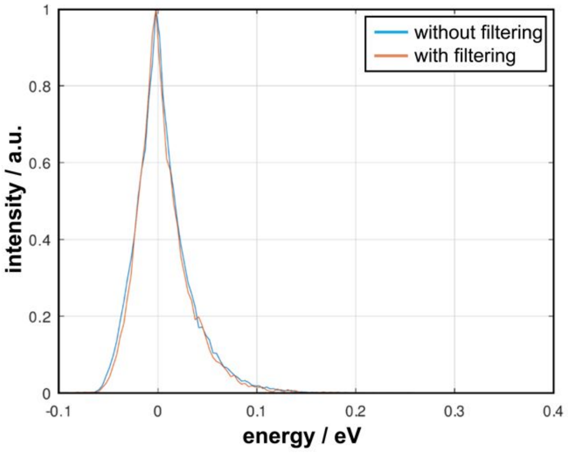

Figure S4 Comparison between the same simulation done with and without reducing the collision points via filtering;

The linearity of the ion energy measurement is shown in Figure S5. The measured peak ion energies of singly charged ions scale very linearly with the ionisation chamber voltage of the EI source in the range of -10 V to +100 V (linear least squares fit: $y = 0.998 \times x - 0.16$, $R^2 = 1$). Note that here the orifice was biased by -20 V relative to the ionisation chamber, resulting in apparent negative energies if the ionsation chamber is set to a negative potential.

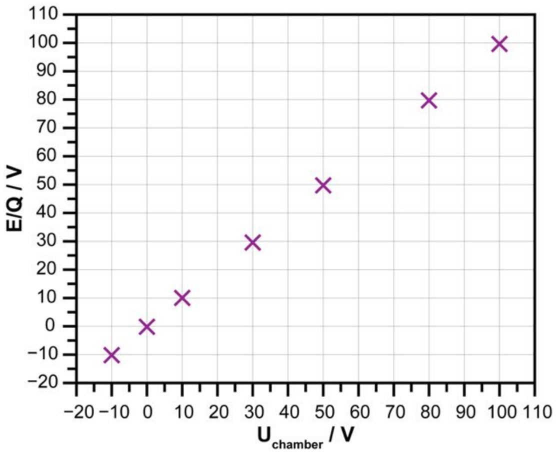

Figure S5 Measured maximum of the ion energy distribution E/Q measured for different voltages of the ionisation chamber $U_{chamber}$;

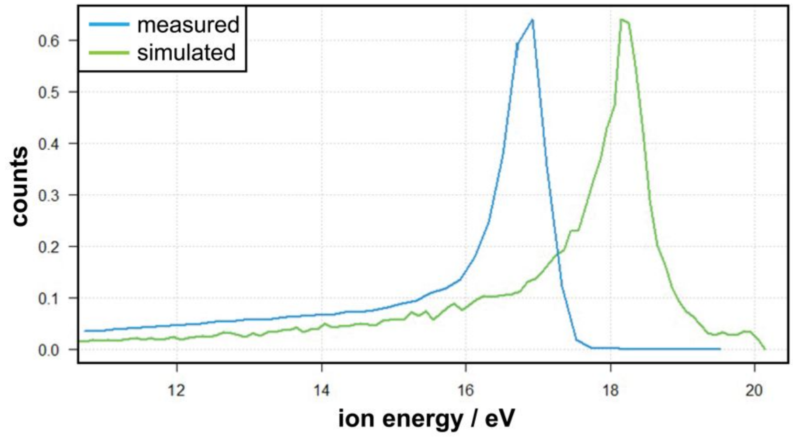

Figure S6 Comparison of measured and simulated ion energies produced by the EI source at 20 V $U_{Chamber}$ and 0 V $U_{extractor}$: ESA : 25eV, exit slit 1 mm;

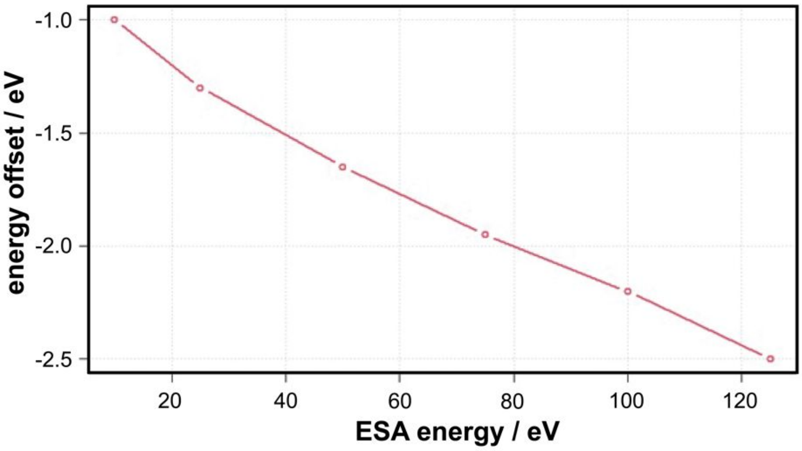

Figure S7 Energy calibration, where the ESA energy depends on the strength of the sector field and corresponds to the energy-per-charge unit of the ions passing the filter, and the energy offset is the difference between the energy expected based on simulations and the set voltage in the acceleration region;

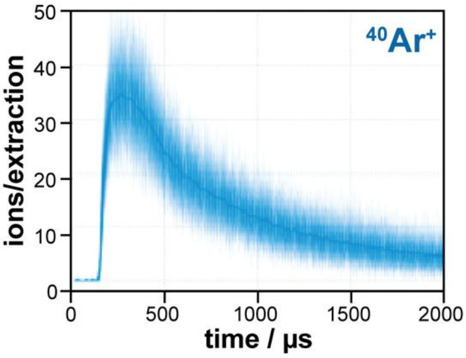

Figure S8 Single pulse analysis of argon during zirconium HiPIMS, measurement initiated 20 µs before the start of the pulse;

2. Applications

Figure S8 shows another interesting feature of the E-ToFMS mass spectrum acquired during HiPIMS discharge using the high-speed steel target. The mass spectrum displays the expected Mo^{+} and W^{2+} ions in the *m/Q* range. The mass resolution is not sufficient to clearly differentiate

between the contributions of $^{184}W^{2+}$ and $^{92}Mo^{+}$, having the same nominal mass, while $^{183}W^{2+}$ and $^{186}W^{2+}$ can be easily identified as distinct peaks at 91.5 Th and 93 Th, respectively.

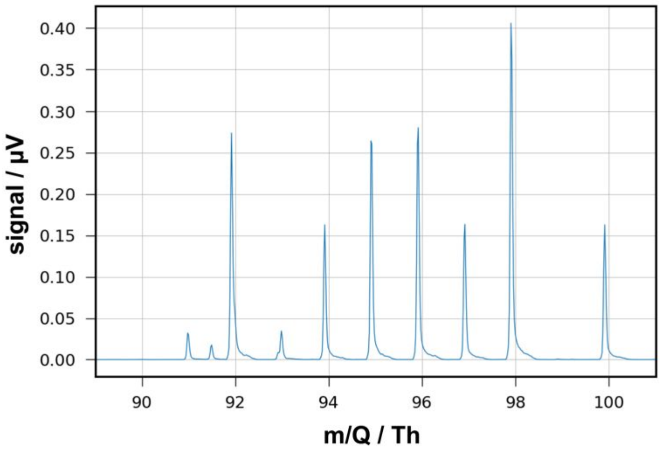

Figure S9 Example mass spectrum showing the range where Mo^{+} and Wo^{2+} isotopes overlap;
